# Central Dental Association of Northern New Jersey

**Published:** 1896-08

**Authors:** H. S. Sutphen

**Affiliations:** Secretary


### CENTRAL DENTAL ASSOCIATION OF NORTHERN NEW JERSEY.

A REGULAR meeting of the Association was held on Monday evening, March 16, 1896, in the parlors of Mr. Simon Davis, Broad Street, Newark, the President, Dr. Walter Woolsey, in the chair.

Present, the following members: Drs. Adams, Adelberg, Barlow, Brown, Crater, Fish, Gregory, Holbrook, Hoblitzell, Hardy, Hane, Hindle, Iredell, Linstead, Meeker, Riley, Rosenthal, Stevens, Sutphen, Wilson, Woolsey.

The minutes of the last meeting and of the annual meeting were read and approved.

The following-named gentlemen were proposed for membership, and the proposals referred to the Executive Committee: K. B. Osmun, of Summit, sponsors Dr. F. C. Barlow and Dr. C. A. Meeker; Julius Peters, of Jersey City, sponsors Dr. C. A. Meeker and Dr. C. W. Hoblitzell; William B. Martin, of Elizabeth, sponsors Dr. Oscar Adelberg and Dr. G. Carleton Brown.

Dr. Meeker read communications from Dr. F. A. Shotwell, of Rogersville, Tenn., and Mr. D. W. Craig, of New York.

*Dr. Meeker.*—Mr. President, I have before me the stenographer's report of the toasts and responses at our annual dinner. The response of Dr. Jack is one of the best expositions of the results of the work of the Boards of Examiners in the United States that I have ever read. The response of Mr. Guy, the editor of the *Electrical Review*, is also of more value than any reports that I have read in the magazines that are devoted to electrical science; and I move you, Mr. President and members of the society, that this report be printed in pamphlet form for circulation among our members and the gentlemen who responded to the toasts.

The motion to print was carried.

*The President.*—Gentlemen, we will now listen to the paper of the evening. I have the pleasure of introducing Wendell C. Phillips, M.D., of New York, who will make some remarks upon "The Principles and Applications of Cataphoresis."

(For Dr. Phillips's paper, see page 418.)

The President then called upon Dr. Hodson to open the discussion.

DISCUSSION.

*Dr. Hodson.*—Mr. President and gentlemen, I have nothing more to say than that I have seen the effects of cataphoresis upon patients, and the effect has seemed wonderful to me. I myself do not use it, and I can speak somewhat as an outsider. I saw the operation upon Dr. Cady, I think it was, where the application of a cold remedy on cotton was crucifying to him, yet after some minutes of the application by Dr. Gillett, under rather unfortunate circumstances, indeed, the effect was really magical. I have seen other operations done in the same way and with the same effects, and it seems to me that not only cataphoresis but this volt-selector for its application are a success.

*The President.*—Dr. Evans, have you something to say on this subject?

*Dr. W. G. Evans.*—In connection with cataphoresis I have really had a large experience, having had the privilege of being with the men who did the first and probably the last work that has been done in that direction. The present outlook is good, and I may say that the best promise of the near future in dental work is in the sterilization of decayed roots with solutions of pyrozone. That will, in all probability, be made a very practical thing as a consequence of the introduction of cataphoresis and the fractional volt-selector. I may also say that here to-night I have received a suggestion which is of great importance, and that is that a combination of arsenous acid and cocaine may be driven into the living pulp, not only to obtund the sensitiveness of the shrinking nerves, but also to permit you to assume the responsibility either of extirpating the pulp or of having it die without pain. It seems to me that really there is something of very great importance in that suggestion.

The question of uric acid is one that we are likely to get some light on from the essayist. I would like to ask Dr. Phillips if he has, in the treatment of the throat, made any observations other than those he has mentioned to-night with regard to the condition of the teeth in patients with gouty manifestations of the breathing passages?

Some of the best writers now claim that uric acid is to be credited with many of the pathological conditions of the teeth, and it seems to me that Dr. Phillips might give us valuable information about the throat and nose in that connection. I make this suggestion because I think he is in the line of bringing to the front that which you need most in the way of diagnosis.

*Dr. Phillips.*—With regard to the question raised by Dr. Evans as to gouty diathesis, I cannot say that I have made accurate investigations in the direction of his inquiry, but I shall return home to study the history of the teeth in connection with these things, and in the course of two or three years I will come back to the New Jersey Central Association and give you a report. I am sure that a rheumatic diathesis is often associated with affections of the nose and throat. I have one case of acute tonsillitis that has a rheumatic condition back of it, and I am curing the acute tonsillitis solely by the use of antirheumatic remedies. I think we are in the line of making some discoveries that will bring about good results in the future.

Dr. Phillips made some demonstrations with the electric mouth-lamp.

*Dr. Hahne.*—Mr. President, I think that I will only express the feeling of all present here when I say that the paper is one of the best lectures that has been given before our society, and I move a vote of thanks to Dr. Phillips. I also move that he be made an honorary member of our society.

The motion was seconded by Dr. Meeker and carried unanimously.

Adjourned.

---

A REGULAR meeting of the Association was held on Monday evening, April 20, 1896, in the parlors of Mr. Simon Davis, 943 Broad Street, Newark, after the usual banquet, the President, Dr. Walter Woolsey, in the chair.

The minutes of the last regular meeting were read, and after amendment approved.

The secretary read a paper by Mr. G. H. Guy, editor of the *Electrical Review*, entitled "The Future of Dentistry electrically considered."

(For Mr. Guy's paper, see page 491.)

DISCUSSION.

*Dr. Sanger.*—Mr. President and Gentlemen,—Your treasurer, in asking me to open the discussion of this paper, exhibited a most deplorable lack of consideration for your feelings.

I never heard of the paper before this evening, and my understanding of its contents is therefore limited to the knowledge I was able to gain of it as it was being read by the secretary. Under these circumstances any adequate discussion of the paper is impossible and an attempt to discuss it is dangerously near the absurd.

The subject is of rapidly increasing importance in medicine and surgery, and no less so in dentistry, which is a part of the science and art of preserving health and healing disease. I will not thereore attempt the impossible, and to avoid the absurd will limit my remarks to a few suggestions as to possibilities in this direction.

Some of us, who had the pleasure of being the guests of the Brooklyn Society not long ago, heard a concise and intelligent discussion of some interesting experiments in using electricity, and especially with regard to cataphoresis.

The thought came to me there that certain well-known psychological laws, acting under these circumstances, might cause some of the results in obtunding sensitive dentine that had been credited to cataphoresis. The effect of suggestion has been shown again and again in lessening or removing the sensation of pain. And in the present attitude of the average mind towards electricity and its wonderful power, the condition of receptivity of suggestion in connection with electrical appliances is extremely favorable for obtaining the best results. Auto-suggestion will have a free field, and the mental state of the operator will influence results.

The action of the electric current in cataphoresis is more satisfactorily shown, it seems to me, in bleaching teeth. Those who have tried this claim that the bleaching power of electrozone and pyrozone is increased by using with them the electric current.

I am not prepared to say how far this is true, although I have made some interesting experiments in this direction. Not having reached a definite conclusion as yet, I do not wish to make any detailed statement of them.

There are gentlemen here who have followed this subject from the beginning, and I cannot do better than give place to them.

*The President.*—I will call upon Mr. Evans, of New York, to speak upon this subject.

*Mr. W. J. Evans.*—Gentlemen, I feel much indebted for the honor which you confer on me on this occasion; but when Dr. Sanger says of me, in connection with others present, that I am one of those who know all about the subject under discussion, it is most embarrassing to me, and I want to deny the allegation at once.

There are many things in connection with this subject which, considered from the stand-point of physics, chemistry, or therapeutics, will give you facts which will yet be a portion of medical history, and which will mark this decade as an important epoch in dental surgery. Cataphoresis is a subject with which I have been familar for some considerable time. In regard to the particular branch of the surgical profession which you practise, Dr. Sanger has very rightly said that cataphoresis can be no bugaboo when we have the bleaching of teeth as a result of its influence.

It will take us some considerable time before we understand all the vagaries that there are in connection with cataphoresis. There are many things maturing hopefully in this connection. Some reference has been made to glycerin in connection with cataphoresis. Glycerin was never intended by Professor Morton to be used in connection with guaia-cocaine and the electric current. What he suggested was that this combination might be applied to the gums. He has now given up entirely the use of glycerin and crude guaiacol, for the reason that guaiacol as formerly obtained was not a preparation from which the best results could be secured. It did have a certain escharotic effect, and a very decidedly bad odor. Recent experiments in the laboratory of McKesson and Robbins have proved that a compound without odor, equally effective, is possible. Investigations of Professor Prescott and others are now in progress which will give you within a few weeks a very important improvement in guaia-cocaine. In connection with the sterilization of the roots of teeth, it has been proved to be a fact that such sterilizers as pyrozone, and other things of that character, can be driven into a porous structure by the aid of the electric current; and the sterilization of the roots of teeth is a thing which will occupy the attention of some of the very best men in your profession.

I have recently had very great privileges in connection with the X-ray. I have been a great deal with Professor Morton, and I can promise those of you who attend the lecture which he is to give before the New York Odontological Society next Friday a very rare treat; and I think I may ask as many of you as have not received a card of invitation to come to that meeting, because I know, from the character of the men who issue those cards, and the enterprise of the society having this lecture under their auspices, that you will have a hearty welcome. I know they wish to have, and expect, a large number present, for they have taken a large room in the Academy of Medicine instead of the smaller room usually occupied by the Odontological Society. I can assure you that you will be able to see there some magnificent stereopticon views, photographs which will show you not only the skulls of the dead, but of the living as well. You will see on the platform that evening a handsome young gentleman, a dental student, and then you will see exhibited on the same platform, absolutely without any embellishment, his skull; you will see each particular tooth represented; you will see its pulp-chamber, without a filling, represented in lines, which indicate that the centre has less osseous density than the outside of the tooth. You will be able to see radiographs, or scotographs,—referring to darkness and shadow; you will see that shadowgraphs indicate to you any deformity of the roots of the teeth, or any teeth that have not come into position in the arch, or any osseous deformity which may take place in the jaw; and you will be very much surprised to find that, regardless of the fact that bullets can be shown in the body, copper washers placed at the side of the skull will show through the skull and its contents.

They will show you photographs of the whole of the skull, and all of the teeth in it can be perfectly illustrated by the influence of the X-ray on a plate. I only say this to show you that the skull can be penetrated by this ray, and that therefore the X-ray seems to be a very important thing in dentistry. Lost tips of drills will be shown as clearly as you can see the fish-hook in a trout without seeing the artificial fly which the fish has swallowed. You may be able to see any little differences in the osseous structure of the mouth, as well as you can see the most delicate lines of a fish's skeleton. Bullets lodged in the body may be shown by the cathode ray, and I predict that you will be able to see many things in connection with malpractice in dentistry which have not yet been seen. I congratulate you that you are in that department of surgery, because I have already been told by Professor Morton that the X-ray offers really more to the dentist than it does to the general surgeon. I thank you, Mr. President, for calling upon me, and for your kind attention.

*The President.*—I will ask Mr. Woolf if he cannot give us some idea in regard to the bleaching of teeth by means of electricity.

*Mr. A. E. Woolf.*—I thank you, Mr. President, for the honor you do me; but, being much devoted to other things in my laboratory, I have not been able to give as much time and attention to the action of electrolysis in the decomposition of organic matter in connection with dentistry as I would have liked to; and, having many very warm friends in the dental profession, I have to acknowledge negligence on my part in the past, but I hope it will be made up in the future. The only thought that I can suggest which might possibly be of interest to you would be the action of electrolysis and the part it plays in dentistry. For example, take a saline solution and apply it to a cavity containing organic or decomposed matter, and apply the electric current, the action would be the decomposition of the electrolite, electrolysis taking place. At the anode or positive pole,—which you would probably insert in the cavity,—in the decomposition of the chloride you would liberate the chlorine from the chloride, and oxygen from the water, both being positive elements. The action of the chlorine would be to combine with the hydrogen of the decomposed matter, leaving the decomposed matter as it existed minus one of its elements. The action of the oxygen would be to oxidize the remaining portion. In the case of peroxide of hydrogen coming in contact with organic matter, decomposition takes place, and one atom of oxygen is liberated. In that case you merely get an oxidizing action. The action of oxygen plus chlorine would be the more rapid decomposition of the organic matter. With a proper current and a proper electrolite you would have a very rapid decomposition of the coloring matter. I cannot speak from practical experience, I can only speak from theory, and the theory certainly would be that there would be complete decomposition of the coloring matter. I have not gone sufficiently deep into the subject to be able to add much to your information, but I will devote some time to it in my laboratory, and on some future occasion I hope to give you something that possibly may be of interest to you, in this connection. In the mean time allow me to thank you very much for your attention.

*The President.*—There are some gentlemen present who have had experience with cataphoresis; we would like to hear from them on this subject. Dr. Palmer, I think, can tell us something about it.

*Dr. Palmer.*—Mr. President, I have had some experience with the use of the electric current, but not to the extent that I am prepared to either read a paper or make any extended remarks upon the subject. On two or three occasions at clinics I have used the Wheeler volt-selector in obtunding sensitive dentine with remarkable results, even better results at clinics than I have had in my own office. Whether that was due to the fact that my friend Evans was present, or because the preparation of cocaine that we used was fresher, I cannot say; but in every case where I have used the electric current in this way it has been very successful. In my experiments on sensitive dentine in my own practice I have been endeavoring to reduce the length of time required to obtain the anæsthetic effect, and I have succeeded in obtaining complete anæsthesia in a much shorter time than has been indicated by Dr. Gillett, of Newport, who is, I presume, the best authority in dental circles on such matters. As to the use of the electric current for bleaching teeth, I would not, I think, in the presence of Dr. Meeker be expected to say very much, but from the experience I have had I think I should support him in anything he might say upon that point.

*Dr. Brown.*—What is your minimum time for obtaining complete anæsthesia in a buccal cavity?

*Dr. Palmer.*—Five minutes; in a lower bicuspid, one-third of the crown being denuded from some cause, and the whole grinding surface being particularly sensitive, there being also a cavity in the posterior portion of the tooth which connected with this abraded surface on the crown. In five minutes time it was so completely anæsthetized that I was able to use the bur without any pain whatever.

*Dr. Brown.*—What preparation did you use?

*Dr. Palmer.*—A twenty-per-cent. solution of cocaine.

*Dr. Brown.*—In what medium?

*Dr. Palmer.*—A very small dilution; using, of course, the electric current. In most of my experiments I have used a twenty-per-cent. solution of guaia-cocaine, to which Mr. Evans has alluded to-night.

*Dr. Brown.*—Mr. President, it is certainly an important thing to reduce the time required in the cataphoric obtunding process. I have been working in this direction since the Baltimore meeting, and since I received my machine, and I must say that I believe we will be able to reduce the time so as to make it possible to use cataphoresis in our work. When it was a matter of twenty-five or thirty minutes time in the obtunding of every cavity it really was almost beyond consideration; but I succeeded a few days ago in securing complete anæsthesia in one minute and a half, in a cavity similar to the one spoken of by Dr. Palmer, but not as extensive situated in a lower canine, and which extended under the free margin of the gum quite a distance. I applied the solution with the electric current for a minute and a half with the idea of first obtunding the gum and getting a superficial effect on the tooth so that I could apply a clamp, expecting to apply it the second time for a deeper effect after placing on the clamp; but before doing that I thought I would see how far the effect had penetrated into the tooth, and I found all sensitiveness had been taken away. I proceeded to excavate the denuded surface and made my retaining points and grooves, cutting a little farther than was really necessary; and this effect was obtained in one minute and a half. I have my assistant stand by me and take the time by the watch in every case that I do since I commenced this series of experiments. And the preparation I used was not guaia-cocaine. It was a preparation which any gentleman can make in his office: simply fifteen per cent. of cocaine in electrozone. What it makes I do not know. I asked Mr. Woolf that question to-day, and he gave me an answer that I could not quite understand. However, this combination certainly makes the preparation that we are after. The first result of mixing them together is that the solution turns a creamy white color for a moment, then turns to a deep yellow. What it is I do not know, but the effect of the preparation—and I have used it right alongside of other preparations—is remarkable. I only hope that you gentlemen will experiment on that and see what results you can get in the same line. In other cases than the one I speak of I have had complete anæsthesia in a somewhat longer time,—three and a half to five minutes. In one case, where I used a salt solution, the time occupied was twenty minutes, while in another and similar case I used an electrozone solution and obtained a better result in five minutes.

*Dr. Palmer.*—Mr. President, did I understand Dr. Brown to say that in the case where the anæsthesia occupied a minute and a half he applied the obtunding solution to the gum?

*Dr. Brown.*—To both the gum and the tooth.

*Dr. Palmer.*—Did you apply it after putting on the rubber dam?

*Dr. Brown.*—No.

*Dr. Palmer.*—How far below the margin of the gum did it extend?

*Dr. Brown.*—About half an inch.

*Dr. Palmer.*—It has been suggested to me that in the case of incisor teeth, particularly, it should be applied to the mucous surface before putting on the rubber dam so as to reach the nerve-supply of the tooth; it being claimed that in that way you anæsthetize the tooth quicker and the effect is more lasting.

*Dr. Brown.*—After the first series of experiments that I made I was talking with Mr. Wheeler about the results, and he could not understand the matter, because he thought that when the application was made to the membrane it would be carried off by the current or the circulation through the membrane, and would not have the proper effect on the tooth, but we secure the result in spite of theory. I do not pretend to know scientifically why it is.

*Dr. Richards.*—Mr. President, Dr. Meeker has been experimenting in this line to quite an extent, and I have no doubt the members present would be glad to hear from Dr. Meeker the result of his experience.

*Dr. Meeker.*—Mr. President, I would like to speak first about Dr. Guy's paper. I think it is one of the best papers, and from a lay member, that this society has ever heard on the subject of cataphoresis or of electricity; it is the most easily assimilable; and I am glad it has been read to-night. As the president has stated, it was to have been read at our annual meeting, but it did not reach me until about half-past eleven that night, so, of course, it could not be read then, and Dr. Phillips answered to the toast to which Mr. Guy was to respond. I have no doubt that next year we will be able to announce for this meeting that we will have Nikola Tesla here. Things are in such condition now that I think he will be with us, although I cannot positively say that he will.

In regard to the bleaching of teeth by means of cataphoresis, or by the usual dry heat method, I have been doing that now for about four years; and Sunday before last I looked over my list of cases, and found that in those four years but three cases have returned to me out of many hundreds that I have bleached. I think that a pretty good average. I have bleached for my own patients and for my friends in the profession here in Newark, and in New York, and other cities. My experience with hot and dry air for this purpose has been perfectly satisfactory so far. Among the cases that came back was a central incisor that had a How pin in it which had turned green, and I could not get it white. In the other two cases I think the canaliculi of the teeth above the gum were so permeated by decomposed pulp-matter that it was impossible to bleach the teeth; the coloring-matter seemed to run down again after they had been bleached, and again turned them dark. Those I have cut off and replaced by artificial crowns. In my experience with cataphoresis, so far I have been quite successful. To-morrow I have two patients promised me by Dr. Walker, at a clinic before the Odontological Society, at the Academy of Medicine, New York. They are said to be very black; I have not seen them but I have no hesitation in saying, from my previous experience, that I will bleach them white, provided there is no inflammatory action going on at the apices of the roots. I have not been able to use the Wheeler volt-selector. I have an eight-cell battery, and when I use electricity for bleaching, I use the full strength of the eight cells, with an aqueous solution of pyrozone. I have not yet used electrozone for this purpose. Pyrozone has been perfectly satisfactory as a bleaching agent so far. Electrozone may be theoretically preferable. I have not tried it, but I am willing to try it and see whether it is good. The theory is all right. In regard to the use of electrozone with cocaine I will say that Dr. Brown informed me in regard to it, and on Sunday I prepared some for use in the morning, having a case to treat, a lateral incisor, which I think I dreaded just as much as the patient for whom I was to fill the tooth. She was of a very nervous temperament, and I felt that I could not, without giving her a great deal of pain, excavate the tooth so as to fill it. So I applied Dr. Brown's preparation of electrozone and cocaine for about fifteen minutes. Then I opened up the pulp-canal with a Donaldson broach, and excavated the tooth without causing any pain whatever. I filled the tooth, and the lady went away rejoicing.

*Dr. Sanger.*—When Dr. Brown was speaking of using a cocaine solution on the gum, the thought occurred to me whether he observed the toxic effect of cocaine, whether the cocaine was taken into the system through the circulation, as it would be if introduced by means of a hypodermic syringe, and with the same danger of toxic effect.

*Dr. Brown.*—I will say that this is a point in which I am most deeply interested, and I am not yet ready to give any positive opinion on it. I asked a number of authorities before starting, how it would work in that respect, and the consensus of opinion seemed to be that the action of the cocaine was limited, and that the toxic effect, if it occurred at all, did not take place until after the cataphoric effect was over. I think the fact of the matter is that you never can tell when you will have the toxic effect until the patient exhibits it. For instance: I know a man who is the most absolute picture of health that you can imagine; you would think he could take anything from a gallon of whiskey to a quart of cocaine, and never have it affect him; yet last summer he went to a dentist, down in one of the sea-shore resorts, to have a wisdom-tooth, erupting with difficulty, attended to; they wanted to lance it, but the gum was so inflamed that they could not touch it, and a four-per-cent. solution of cocaine was injected. The relief was instantaneous, but when the gentleman undertook to leave the chair he dropped to the floor and was unconscious for three hours. No one would have expected a toxic effect in that gentleman. It is not like administering an anæsthetic where you can tell by watching the heart of the patient through the pulse, if you are careful. I have carefully watched my cases, and I have not noticed any toxic effect or variation of the heart's action, nor have I as yet seen the slightest indication of any such change; but I think it has not been used long enough for one to form an opinion on that point. I would suggest to any gentleman using it that he carefully watch the pulse of the patient. I never use cocaine hypodermically.

*Dr. Osmun.*—Mr. President, all the talk to-night has been from the side of the operator; I want to talk to you now from the side of the victim. Two weeks ago, Saturday evening, Dr. Van Woert applied the cataphoric current on a lower canine—a buccal cervical cavity—of mine, that was so intensely sensitive that I could not brush it, the slightest touch of the tooth-brush gave it pain. Dr. Van

Woert made the application for about fifteen minutes on the gum tissue and on the tooth itself, with a thirty-per-cent. aqueous solution of cocaine. He experimented a little with the current, starting with a low voltage at first, and there was no pain whatever. Then he turned on four or five times as much, which gave acute pain and was unbearable. He then turned off the current and renewed it gradually, a little at a time, and it caused no pain whatever; and the tooth became perfectly insensible. It was not filled at that time, but was probed deeply, and the gum was also probed, and both were absolutely painless. The gratifying part is the fact that the tooth has been absolutely painless ever since. I have sent an excavator in every day without finding any sensitiveness; while outside of the zone of cataphoric application the tooth is just as sensitive as though there never was any cocaine used. That would seem to prove conclusively that the cocaine did not enter the circulation, or was not carried to any extent beyond the point at which it was applied. I think that is a very important fact, because if you can produce local insensibility of the tissue and retain that insensibility after the filling has been inserted, it is a very great thing. You know that usually there is a condition of hypersensitiveness or pain for two or three days to five or six weeks after such operations.

*Dr. Riley.*—I would like to ask Dr. Woolf to give us the chemical change that has taken place when electrozone and cocaine are used in that way.

*Dr. Woolf.*—I became interested for the first time in this matter of cocaine and other materials used in dentistry to-day when I happened to wander into Dr. Brown's office.

What I learned from Dr. Brown of the results of his experiments struck me as very peculiar, and I am free to confess that I cannot possibly understand under what conditions the action of this material in conjunction with cocaine should produce the wonderful results that Dr. Brown has described to me. That the electric current will pass through the tissues; that electrolysis, would possibly or positively take place in the passage of the current, and that the positive and negative elements would be liberated in this electrolysis is easy enough for all of us to understand; but how the electric current can convey or carry with it the properties contained in cocaine I am free to confess that I am at a loss to comprehend. The question is so remarkably interesting to me, and so undecided in my mind, that it will be a source of pleasure to me to delve more deeply into the subject; and, without endeavoring to appear at all presumptive to the Association, I will communicate to you at the earliest possible date the result of my experimentation. There is, in my mind, no possible way, by passing an electric current through the sensitive tissue, of carrying the properties of cocaine with that current. It may be possible to intensify, to set up an activity in the membrane, and so make the membrane more sensitive to the action of cocaine; it may be possible that the cocaine would in some instances have its effect regardless of the electric current, and it may be possible that the electric current would act without the cocaine; but the fact that Dr. Brown tried cocaine alone, and tried electrolysis alone, and tried the different elements of the material alone, and in none of these instances did he get the results that he obtained from making the experiments with cocaine and the electric current, left me undecided concerning what the action was.

*Dr. Brown.*—As to this combination, I do not claim to have made, personally, any discovery. Dr. Van Woert suggested salt water; that brought up the idea of using electrozone with cocaine. As Dr. Osmun has talked from the stand-point of the patient or the victim, I think I will tell something about the patient too. To a considerable extent our work in this line is experimental, we do not know what we are doing. I used with the most perfect results, requiring too long a time, however, a saline solution on two superior incisors, and the patient was delighted. I did not see him for a week after, and when I did he said he had not recovered from the cataphoresis. He said he felt nothing until about the end of twenty-four hours, when he commenced to have a queer feeling in those teeth, and he had that for three days afterwards. He said it was the same sensation that one feels when we raise the current of electricity. And right there was another peculiar point. On one of the teeth I used fifteen volts, and on the other I used forty volts without having a particle of bad effect. In both cases it was successful as far as the clinical work went. I have examined the teeth since, and they are in perfect condition. They are alive, there is no hypersensitiveness to heat or cold, and the filling is all right; but for three days they were very uncomfortable. We may as well report those cases as the others.

*The President.*—Dr. Adelberg has had some experience with cataphoresis, I will call upon him.

*Dr. Adelberg.*—My experience, Mr. President, has been very limited, and there is so much uncertainty about it that it is hardly worth while reporting. I had two cases in one day that I treated cataphorically, almost the same kind of cavities in each case; in one case I used the saline solution in connection with cocaine, and obtained perfect anæsthesia in eight minutes with an application of thirty-four volts, and prepared the cavities without a particle of pain. In the other case, the same afternoon, I could not possibly use over six volts. I tried it for fifteen or twenty minutes without success. So my experience so far is very experimental.

*Dr. Brown.*—I believe the president has had some experience; I think we might hear from him.

The vice-president, Dr. Fish, takes the chair.

*Dr. Woolsey.*—In the two weeks that I have had my machine I have made some use of it and had some good results. I had a peculiar case on Monday morning, a young lady not in very good health, about sixteen years of age, and it seemed almost impossible to obtain the desired result from cataphoresis. I was working upon a central incisor; the current caused pain, and it seemed impossible to get the voltage up to ten volts. The next day, Tuesday, I tried it on the same patient, in a large approximal cavity in a bicuspid, and it was perfectly successful. I was able to raise the voltage in twelve minutes to about twenty-five volts, and was able to work on the tooth without causing a particle of pain. The patient is very sensitive to the use of the bur, generally objects to its use. In this case I wanted to place in a gold filling, so there was more cutting required than in other cases. In some cases I have had good results, but it has taken a considerable length of time, and that has been discouraging. In most of those operated upon it has taken from twelve to fifteen minutes. In one case by applying the electrode to the neck of the tooth instead of in the cavity I seemed to get a better result. Whether there is anything in that or not I do not know, but it seemed so in that case. I find that by mixing my cocaine solution at the time of using the effect is better.

The president resumes the chair.

On motion of Dr. Richards the subject was passed.

*Dr. Meeker.*—I would like to announce that Dr. Twilley, of Baltimore, will give us a paper in June, and Mr. Davis has promised to have an electric fan here to keep us cool. I will also say that any gentleman who would like to go on the excursion to the proposed colony on the Delaware on the 29th of May, to spend Decoration Day and Sunday, will please leave their names with me the day before the 29th.

Adjourned.

H. S. Sutphen, D.D.S.,
*Secretary.*

